# Radiotherapy with apatinib for recurrence of malignant phyllodes tumor of the breast: A case report

**DOI:** 10.1097/MD.0000000000018808

**Published:** 2020-01-17

**Authors:** Hong Wu, Lei Li, Jing Yang, Chen Guo, Weiming Zhang, Hui Wang

**Affiliations:** a Department of Oncology; b Department of Orthopedic trauma, Affiliated Hospital of Binzhou Medical University, Binzhou, China.

**Keywords:** apatinib, breast, case report, phyllodes tumor

## Abstract

**Rationale:**

Malignant phyllodes tumors of the breast are rare, and there are currently no guidelines and a large number of clinical trials to guide the treatment of recurrence tumor. Here we reported a case of radiotherapy with apatinib for the treatment of recurrent malignant phyllodes tumor of the breast.

**Patient concerns:**

A 58-year-old patient with massive breast mass was admitted to our hospital. Two months after surgical treatment, the tumor recurred in the chest wall scar.

**Diagnoses:**

The histopathologic diagnoses was right breast malignant phyllodes tumor with chondrosarcomas and osteosarcomas in some areas.

**Interventions:**

The patient was first treated with surgery. Malignant phyllodes tumor recurred in the chest wall two months after surgery and was treated with radiotherapy and apatinib.

**Outcomes:**

With surgery, radiotherapy and apatinib treatment, the patient still died within several months.

**Lessons:**

Apatinib and radiotherapy failed to obtain good therapeutic effect in the recurrence of breast malignant phyllodes tumor in this case.

## 1 Introduction

Phyllodes tumors are rare fibroepithelial neoplasms breast tumors and are found in account for only 0.3 to 0.5% of all breast tumors.^[1]^ The WHO classify Phyllodes tumors as benign, borderline, and malignant that base on stromal patterns of cellularity, nuclear atypia, mitotic activity, heterologous stromal differentiation, Phyllodes tumors, stromal hypercellularity and tumor margin appearance.^[2]^ The majority of phyllodes tumors occur in women between 35 and 55.^[3]^ The pathogenesis of general phyllodes tumors has the following several kinds: endocrine hormone disorder, fibrous adenoma on the basis of progress, race and reproductive lactation and other factors.^[4,5]^ Surgical treatment is preferred for malignant phyllodes breast tumor. However,there are few reports on the sensitivity of radiochemotherapy and other drugs after tumor recurrence.

In this report, we present a rare case of malignant phyllodes tumor that developed on the basis of fibroadenoma and treated it with surgery, radiotherapy and apatinib. But the patient's condition continued to deteriorate rapidly and eventually died within several months.

When the patient's disease worsened, informed written consent was obtained from the patient for publication of this case report.

## 2 Case presentation

A 58-year-old female patient was admitted to our hospital in September, 2018. But the history of breast related diseases started eight years ago. In October 2010, the patient had a painless mass about 0. 5 cm in the upper quadrant of the right breast. Standard mammography examination indicated cystic changes in double breast and nodules in the right breast. The patient underwent a minimally invasive resection of the tumor. Postoperative pathology indicated breast hyperplasia and fibroadenoma. In July 2012, the patient's right breast mass recurred. Right breast mass resection was performed again, and the postoperative pathology was still fibroadenoma of breast.

**Figure 1:**
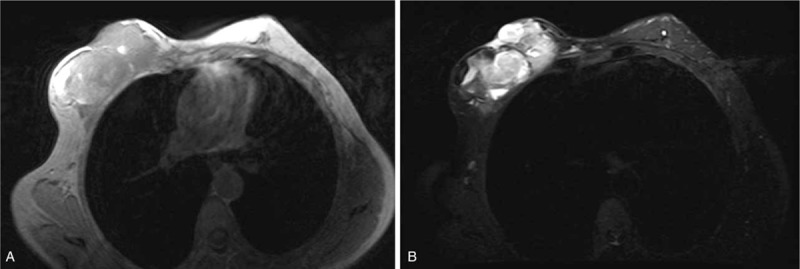
Magnetic resonance imaging findings. A,B Magnetic resonance images showing a large tumor in the right breast.

The recurrence of the right breast mass occurred in June 2013. At that time, the size of the tumor was about 1 × 1 cm, but the patient chose not to have surgery. Five years later, in September 2018, the mass of the right breast increased to about 15 × 10 cm. The pain in the right breast was obvious. The volume of the right breast increased significantly, with high skin tension, local redness and obvious tenderness, occupying most of the breast. Magnetic resonance examination of the breast suggested space-occupying lesions in the right breast, which was considered as breast cancer [BI-RADS category 5] with enlarged lymph nodes in the right axilla (Fig. 1). The patient underwent right breast mass biopsy under ultrasound guidance. Postoperative pathology indicated a right breast phyllodes tumor. Then, the patient underwent surgical treatment, and the surgery was as follows: right breast phyllodes tumor expanded resection + axillary lymph node dissection + free DIEP skin flap repair + fibrous vascular anastomosis×4 +umbilical angioplasty. The histopathologic findings: a right breast malignant phyllodes tumor with chondrosarcomas and osteosarcomas in some areas. No tumor was found in the nipple, incised line and marked incised margin. Immunohistochemistry: CK-,CKT-,Vimentin+. No metastatic tumor was found in the right axillary lymph node (0/27).

**Figure 2:**
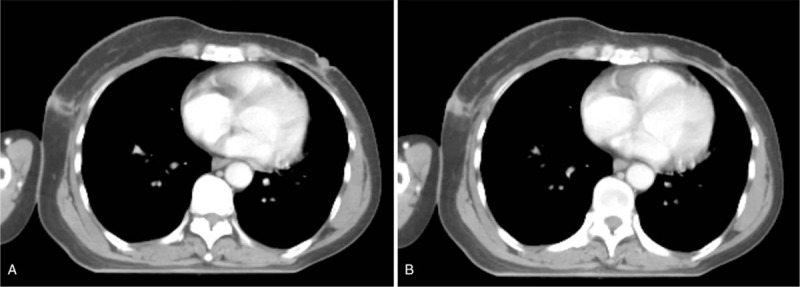
Computed tomographic findings. A,B Computed tomographic images showing the tumor recurred in the scar on the right chest wall.

The patient rested for 2 months after surgery. Then the patient was found to have a 1 × 1 cm nodule in the surgical scar on the right chest wall. The skin at the nodule is reddened without tenderness. Computed tomography (CT) imaging of the chest revealed a small tissue density mass in the right chest wall (Fig. 2). The pathological results of nodular puncture showed malignant tumor, which tended to be phyllodes tumor. The oncologist gave radiotherapy to the right chest with the recurrent nodule. The prescription dose was PTV 60Gy/30 fractions. Because of the patient refused chemotherapy, she was treated with apatinib. The apatinib dose applied was 0. 5 g per day continuously.

**Figure 3:**
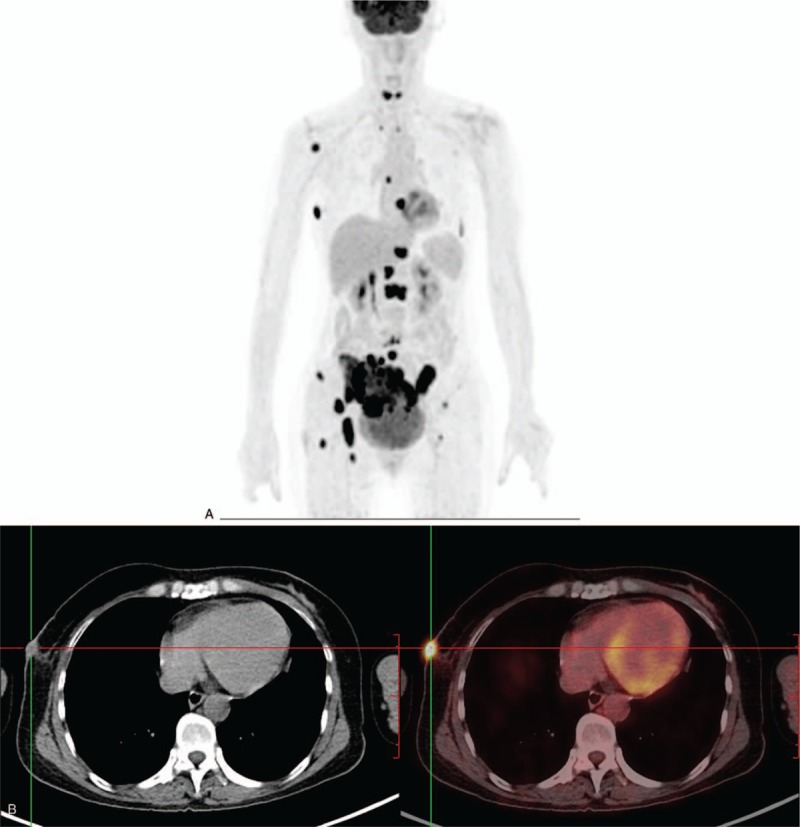
Positron emission tomography imaging findings. A, Positron emission tomography images showing multiple bones metastasis. B, The local activity of the recurrent tumor of chest wall existing after radiotherapy.

Half month after radiotherapy, the patient presented lumbosacral pain. PET-CT showed that the patient had multiple bones metastasis (Fig. 3A). The retraction of the tumor in the radiotherapy area was not obvious (Fig. 3B). Oncologists recommend systemic chemotherapy and radiation therapy for metastatic lesions. The patient refused other treatments and continued to take apatinib. A month later (in March 2019), the patient had frequent seizures at home, combined with headache and mental disorder. In the end, we speculated that she died of brain metastases, brain hernia.

## 3 Discussion

Phyllodes tumor (PT) of the breast is a rare fibroepithelial neoplasm, accounting for 0. 3% to 1% of all breast tumors.^[6]^ PT morphology shows continuity from benign to malignant. Approximately 85% to 90% of phyllodes tumors are benign and 10% to 15% are malignant.^[7]^ Pathologically, these tumors are characterized by mildly increased stromal cellularity and nuclear atypia. Benign PT is difficult to distinguish from fibroadenoma of the breast. Malignant PT is characterized by marked stromal cellularity and nuclear pleomorphism, stromal overgrowth. The presence of heterologous sarcomatous elements (liposarcoma, chondrosarcoma, and osteosarcoma) can be a diagnosis of malignant phyllodes tumor. In this case, the patient developed benign PT into malignant PT over a period of years.

The main characteristics of malignant PT are local recurrence and progression. Surgical excision is an important treatment for PT. Breast-conserving surgery with appropriate margins (≥1 cm) is the preferred primary therapy for PT in the absence of metastatic disease.^[8]^ In the research of Adam et al,^[9]^ they concluded that the surgical margin should be at least 5 mm whatever the grade of PT. Moderate to severe nuclear stromal pleomorphism identified a subgroup of grade 1 PT with a higher rate of recurrence. In this case, due to the large breast tumor, the patient underwent extensive mastectomy and axillary lymph node resection. Currently, there are no clear guidelines for the postoperative adjuvant treatment of PT and no prospective randomized studies are available. The study by Kim et al,^[10]^ they found that although patients with more adverse prognostic factors underwent postoperative radiation therapy (RT), RT groups were not inferior to non-RT group on cancer specific survival regardless of surgery. One meta-analysis described that patients treated with postoperative RT had a lower relative risk of local recurrence than those not receiving postoperative RT, even after margin-negative wide local excision.^[11]^ According to the dose effect curve of tumor, NSD,TDF and other formulas, the relationship between radiotherapy dose and tumor control probability can be explained. Tumor control probability is also related to tumor sensitivity, tumor size and many other factors. Generally, when conventional radiotherapy regimen (2Gy once a day, 5 days a week) is applied, the dose control rate of radiotherapy for subclinical lesions is 45 to 50Gy, while the dose for residual or macroscopic tumors is 60 to 80Gy. Because the patient had recurrence of chest wall (macroscopic tumor), conventional radiotherapy dose of 60Gy was finally adopted.

As we mentioned above the presence of heterologous sarcomatous elements (liposarcoma, chondrosarcoma, and osteosarcoma) can be diagnosed as malignant PT. The patient's postoperative pathology revealed a malignant phyllodes breast tumor with chondrosarcomas and osteosarcomas in some areas. In recent years, some new targeted anti-tumor angiogenesis drugs have been developed and shown good efficacy in sarcomas, such as bevacizumab, sunitinib, regorafenib, sorafenib and pazopanib. According to the clinical results of PALETTE III,^[12]^ pazopanib is an anti-angiogenic drug that can be used for non-resectable or metastatic, progressive soft tissue sarcoma. A total of 369 patients with disease progression after chemotherapy were randomly assigned to either pazopanil or placebo. And it turns out, Pazopanib significantly improved PFS (4. 6 months vs 1. 6 months); *P* < . 0001). However, pazopanib has not yet been approved Chinese patients with sarcoma. Apatinib is also approved as an anti-tumor angiogenic drug for the late stage or metastatic gastric or gastroesophageal junction Adenocarcinoma, which is used at least after failure of second-line chemotherapy drugs. Apatinib is a novel and high selectivity inhibitor of the vascular endothelial growth factor receptor-2 (VEGFR2) tyrosine kinase, which will block the downstream signal transduction of VEGFR2 at the cellular level.^[13]^ We found through evidence-based medicine that apatinib had a good effect in some patients with sarcomas (soft tissue sarcomas, osteosarcomas, etc).^[14,15]^ Zhu et al,^[16]^ they found that apatinib showed good efficacy and acceptable safety in patients with metastatic or recurrent sarcomas. In the 24 patients who met the evaluation criteria, the objective response rate was 33. 3% and clinical benefit rate was as high as 75.0%. The patient herself refused systemic chemotherapy. Because currently no standard treatment is recommended for the recurrence of malignant PT. In view of the chondrosarcoma and osteosarcoma tissue in some areas of the patient's malignant phyllodes tumor of the breast, and some relevant studies^[14,15]^ have shown that apatinib has good efficacy in sarcoma, the patient was tentatively applied apatinib for treatment. But apatinib did not achieve good results in this patient.

As a rare disease with high risk of recurrence or metastasis, malignant phyllodes tumor of the breast has no standard guidelines for postoperative or recurrent treatment. Despite the presence of sarcoma in the pathological results, apatinib and radiotherapy for the treatment of this disease were not effective in this case.

## Author contributions

**Conceptualization:** Lei Li.

**Data curation:** Chen Guo.

**Formal analysis:** Lei Li.

**Investigation:** Jing Yang.

**Methodology:** Hui Wang.

**Project administration:** Weiming Zhang.

**Software:** Chen Guo.

**Supervision:** Hui Wang.

**Writing – original draft:** Hong Wu.

**Writing – review & editing:** Hong Wu.

Hui Wang orcid: 0000-0001-5055-5491.
